# Complete Chloroplast Genome of *Krascheninnikovia ewersmanniana*: Comparative and Phylogenetic Analysis

**DOI:** 10.3390/genes15050546

**Published:** 2024-04-25

**Authors:** Peng Wei, Youzheng Li, Mei Ke, Yurong Hou, Abudureyimu Aikebaier, Zinian Wu

**Affiliations:** 1 Grassland Research Institute, Xinjiang Academy of Animal Sciences, Urumqi 830000, China; km1108@163.com (M.K.); houyurong0994@126.com (Y.H.); 18599083688@163.com (A.A.); 2 College of Grassland Science, Xinjiang Agricultural University, Urumqi 830052, China; lyzheng7@163.com; 3 Institute of Grassland Research, Chinese Academy of Agricultural Sciences, Hohhot 010011, China

**Keywords:** Amaranthaceae, *Krascheninnikovia ewersmanniana*, chloroplast genome, codons, phylogenetic analysis

## Abstract

*Krascheninnikovia ewersmanniana* is a dominant desert shrub in Xinjiang, China, with high economic and ecological value. However, molecular systematics research on *K. ewersmanniana* is lacking. To resolve the genetic composition of *K. ewersmanniana* within Amaranthaceae and its systematic relationship with related genera, we used a second-generation Illumina sequencing system to detect the chloroplast genome of *K. ewersmanniana* and analyze its assembly, annotation, and phylogenetics. Total length of the chloroplast genome of *K. ewersmanniana* reached 152,287 bp, with 84 protein-coding genes, 36 tRNAs, and eight rRNAs. Codon usage analysis showed the majority of codons ending with base A/U. Mononucleotide repeats were the most common (85.42%) of the four identified simple sequence repeats. A comparison with chloroplast genomes of six other Amaranthaceae species indicated contraction and expansion of the inverted repeat boundary region in *K. ewersmanniana*, with some genes (*rps19*, *ndhF*, *ycf1*) differing in length and distribution. Among the seven species, the variation in non-coding regions was greater. Phylogenetic analysis revealed *Krascheninnikovia ceratoides*, *Dysphania ambrosioides*, *Dysphania pumilio*, and *Dysphania botrys* to have a close monophyletic relationship. By sequencing the *K. ewersmanniana* chloroplast genome, this research resolves the relatedness among 35 Amaranthaceae species, providing molecular insights for germplasm utilization, and theoretical support for studying evolutionary relationships.

## 1. Introduction

*K. ewersmanniana* is a perennial, strongly drought-tolerant shrub belonging to the genus Krascheninnikovia in the family Amaranthaceae [1]. In addition, the shrub has tolerance to salt and alkali, cold resistance, and other beneficial characteristics [2,3,4]. This species is widely distributed in China, Kazakhstan, Russia, and Mongolia; in China, *K. ewersmanniana* grows exclusively in the arid desert grasslands of the Xinjiang Altay and Tianshan Mountains [5,6,7]. *K. ewersmanniana* is highly effective as a windbreak and aids in sand fixation and water and soil conservation. It is thereby considered the main species in the arid desert area of Xinjiang and plays an indispensable role in restoration and improvement of grassland vegetation [8]. As well as its significance for ecological construction, *K. ewersmanniana* is a good forage shrub for livestock, with high crude protein content and nutritional value [9]. Since *K. ewersmanniana* plants grow tall, they can be used as a life-saving grass in winter and spring in Xinjiang and play an important role in disaster relief [9]. Therefore, *K. ewersmanniana* has high economic and ecological value and is a valuable germplasm resource. Extensive research on the development and utilization of *K. ewersmanniana* has elucidated its morphology, anatomy, physiological and biochemical characteristics, ecology, and cultivation management technology [10,11,12,13,14]. However, few studies have genetically analyzed *K. ewersmanniana*, particularly to uncover its origin, evolution, and genomics.

Chloroplasts are important organelles for plants that convert light energy into chemical energy [15,16] and have independent genome structures and genetic functions [17,18]. Since chloroplast genomes of angiosperms are mostly maternally inherited, their gene sequences are highly conserved and stable, with unique advantages for determining genus or interspecies relationships and phylogeny; as such, chloroplast genomes play a prominent role in phylogeny, plant taxonomy, and species identification [19]. The double-stranded circular configuration is the most typical structure of chloroplast genes, and is highly conserved. The common plant chloroplast genome is generally 120–160 kb, comprising four different DNA fragments: a large single copy (LSC) region, a small single copy (SSC) region, and two separate inverted repeat (IR) regions (IR a/b, IR a/b) [20,21,22,23]. With advances in science and technology, especially the continuous improvement of sequencing technology, the phylogenetic relationships of various groups can be reconstructed through chloroplast genomics, and the taxonomic status and relationships among different plant taxa can be examined to promote better understanding and utilization of plants. Therefore, the use of chloroplasts to study the origin, structure, and evolution of organelles has received increasing attention, resulting in the sequencing and analysis of a rising number of plant chloroplast genomes.

Previous research on *Krascheninnikovia* plants has primarily focused on their ecological and physiological characteristics, with only a few performing chloroplast genome and phylogenetic analyses. Random amplified polymorphic DNA (RAPD) was used to disclose the genetic diversity of seven *Krascheninnikovia* plant samples and the results revealed that the ecotypes of Ningxia, Xinjiang, desert, and Horqin (Inner Mongolia) were grouped together at the molecular level, and were identified as a northern China species (*K. arborescens*), and has close genetic relationship with *K. latens*, *K. ewersmanniana*, and *K. lanata* are differentiated into two independent species [24]. Liu et al. [25] assembled and annotated the chloroplast genome of *K. ceratoides*, and reported that *K. ceratoides* to be closely related to *Atriplex*, *Chenopodium*, *Dysphania*, and *Suaeda*, which is consistent with studies based on nuclear ribosomal internal transcribed spacer and chloroplast (cp) DNA data. However, the did not conduct analysis to distinguish between species or families. Amaranthaceae, which was a global family, was a transitional group from entomophilous plants to anemophilous. The variability in flowers resulted in mass identification of species or genus and taxonomy. Especially, the Amaranthaceae family has recently been extended to include the Chenopodiaceae family based on morphological and phylogenetic analyses [26]. Moreover, according to the flora of Inner Mongolia, the genus *Ceratoides* (Tourn) Gagnebin was proved invalid and the name was then changed to *Krascheninnikovia* [27]. 

To better synthetically comprehend the *Krascheninnikovia* genus’s origin, evolution, and polygenetic relationships, we aimed to analyze the genetic composition of *K. ewersmanniana*. Second-generation high-throughput sequencing technology and bioinformatic analysis methods were used to sequence, assemble, and annotate the whole genome of *K. ewersmanniana* and resolve sequence variations and structural features. Based on the chloroplast genome information of six Amaranthaceae species and *K. ewersmanniana* published in the NCBI database, we have analyzed and summarized the chloroplast genome structures of related groups, followed by selection of chloroplast genomes of 35 related species to establish a phylogenetic tree with common protein-coding gene (PCG) sequences. The purpose of this study is to investigate the phylogeny of Amaranthaceae plants using the chloroplast genome of *K. ewersmanniana*, and to lay a theoretical foundation for the phylolocation, plant classification, and resource utilization of Amaranthaceae.

## 2. Materials and Methods

### 2.1. Experimental Materials

*K. ewersmanniana* germplasm material was collected from Hutubi County, Xinjiang, China (Xinjiang Ministry of Agriculture of China, E 86°37′, N 44°14′, altitude 504 m). A certified specimen was collected along with the leaves (collector: Youzheng Li). The specimen was identified as *K. ewersmanniana*, a *Krascheninnikovia* plant of family Amaranthaceae, by Mei Ke, a researcher at the Grassland Research Institute of the Xinjiang Academy of Animal Sciences. Specimens were stored in the plant specimen library of the Grassland Research Institute, Xinjiang Academy of Animal Sciences. Seedlings were raised in the Xinjiang Academy of Animal Sciences AI climate chamber. Fresh and young leaves from healthy plants were collected for sequencing.

### 2.2. Genomic DNA Extraction and Sequencing

Genomic DNA was extracted from fresh leaves of plants using cetyltrimethyl ammonium bromide, and the DNA concentration was measured using a NanoDrop Spectrophotometer (Thermo Scientific, Waltham, MA, USA) and a Qubit Fluorometer (Invitrogen, Carlsbad, CA, USA). The Illumina Miseq platform was used to sequence the chloroplast genome of the sample (Personalbio, Shanghai, China).

The data quality control was performed by fastp (v0.19.4) and high-quality sequences were produced. Junction contamination at the 3′ end was removed by Adapter Removal (version 2) [28], and the sliding window method was used to filter the quality. The average Q value of the bases in the window was calculated. If the Q value was <20, the bases in the window were deleted, and if Q was ≥20, sliding was stopped. If the length of any reads in the double-end is ≤50 bp and the number of bases in the double-end is ≥5, the double-end sequence is removed to ensure that the sequences contained in the dataset have sufficient length and quality.

### 2.3. Chloroplast Genome Assembly and Annotation

The plant chloroplast genome was assembled using default parameters of GetOrganelles (version 1.7.5.3) [29]. Chloroplast genome annotation was performed using the Plastid Genome Annotator [30]. Error codons were corrected using Geneious (version 9.0.2) [31]. Chloroplast genome data were submitted to GenBank (accession number: PP191169). Finally, a chloroplast genome map was drawn using Organellar Genome Draw software (OGDRAW, version 1.3.1) [32].

### 2.4. Analysis of Chloroplast Genome Repeats and Codon Usage Preferences

Misa software (version 2.1) was used to predict simple sequence repeats (SSRs) of *K. ewersmanniana* chloroplast genomes, with the minimum number of repetitions for single nucleotide, double nucleotide, trinucleotide, tetranucleotide, pentanucleotide, and hexanucleotide sets as 10, 6, 4, 3, 3, and 3, respectively [33]. The online tool REPuter [34] was used to analyze dispersed repeat, with the Hamming distance and minimum repeat fragment size parameters set to 3 and 30, respectively. The relative synonymous codon usage (RSCU) value was calculated using CodonW software v.1.4.2 [35]. RSCU is the ratio of the actual codon frequency to expected frequency. A RSCU value of 1 depicts codon usage without bias, while RSCU value < 1 indicates its relative rarity and >1 indicates that codon usage is greater than expected [36]. 

### 2.5. Comparison of Chloroplast Genomes

The IRscope online tool was used to analyze the infrared boundary information of the chloroplast genomes of seven Amaranthaceae plants [37]. mVISTA software [38] was used to perform collinearity analysis of the chloroplast genomes of different species, with *Chenopodium quinoa* as a reference.

### 2.6. Phylogenetic Analysis

A phylogenetic tree of 35 species in the Amaranthaceae family was established, in which the annotated *K. ewersmanniana* chloroplast genome was assembled, and the genome sequences of 34 other Amaranthaceae species were downloaded from NCBI (Table S1), with *Oryza sativa* and *Arabidopsis thaliana* as outgroups. Nucleotide sequences corresponding to PCG in the selected genome were connected, and multiple sequence alignments were performed using the MAFFT program [39]. A phylogenetic tree was constructed using the maximum likelihood (ML) algorithm and Bayesian analysis, and the best model was selected using ModelFinder [40]. The ML method uses the software RAxML and selects the nucleotide substitution model as GTR + F + I + G4 [41]. The best-fit GTR + F + I + G4 model was selected using Bayesian imputation in MrBayes v3.2.6 [42].

## 3. Results

### 3.1. Genomic Features of K. ewersmanniana

**Figure 1:**
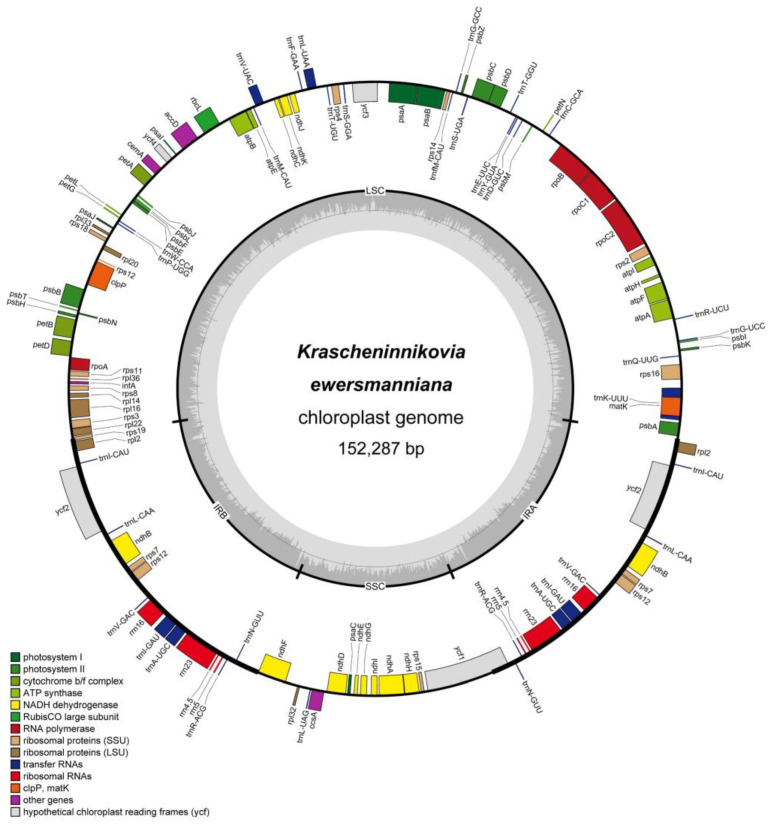
Map of the chloroplast genome structure of *K. ewersmanniana*. Genes drawn in circles are transcribed clockwise, whereas genes outside the circle are transcribed counterclockwise. Different colors are annotated according to the different functions of the genes.

Second-generation Illumina sequencing on the *K. ewersmanniana* chloroplast genome generated 36,875,778 raw reads, of which 712,889 were used for subsequent genome assembly. In this study, we assembled and annotated the complete chloroplast genome of *K. ewersmanniana* for the first time. The chloroplasts of *K. ewersmanniana* comprised four parts (SSC, IRa, LSC, and IRb) and exhibited a unique double-stranded circular structure with a genome size of 152,287 bp (Figure 1). The lengths of the IR, SSC, and LSC sequences were 49,182, 19,007, and 84,098 bp, respectively. The GC levels of the IR, SSC, and LSC regions were 41.99%, 30.58%, and 34.74%, respectively (Table S2).

The annotation results revealed that the *K. ewersmanniana* chloroplast genome can be divided into four categories based on function: self-replication, photosynthesis, other genes, and unknown genes. A total of 128 genes were annotated (84 PCGs, 36 tRNAs, and eight rRNAs). The most abundant were tRNA genes, followed by subunits of the ribosome, with a total of 21 genes, including nine large subunit genes and 15 small subunit genes. Among the tRNA genes, *trnV-GAC*, *trnA-UGC*, *trnL-CAA*, *trnI-CAU*, *trnR-ACG*, *trnN-GUU*, and *trnI-GAU* had two copies each; among the ribosomal protein large and small subunit genes, *rpl2*, *rps7*, and *rps12* had two copies each; among the four rRNA genes, *rrn5S*, *rrn23S*, *rrn16S*, and *rrn4.5S* had two copies each; and the NADH-dehydrogenase gene, *ndhB*, had two copies (Table 1). 

Among the 128 annotated genes, a total of 19 genes contained exons and introns. Among these, only two genes (*ycf3* and *clpP*) had three exons and two introns. The remaining 17 genes, namely *ndhB(2)*, *trnI-GAU(2)*, *petD*, *ndhA*, *atpF*, *petB*, *trnV-UAC*, *rpl16*, *trnA-UGC*, *rpoC1*, *trnR-UCU*, *rps12*, *trnL-UAA*, *trnK-UU*, and *rps16* had two exons and one intron. The two introns of *clpP* were 811 bp and 603 bp long, whereas those of *ycf3* were 779 bp and 805 bp long. Among all genes containing introns, *trnK-UUU* has the longest length of 2493 bp and *rps12* has the shortest length of 543 bp. Among all genes containing exons, *ndhB* had the largest number of exons (1533 bp); 777 bp for exon I and 756 bp for exon II. The three genes with the smallest number of exons were *trnA-UGC*, *trnA-UGC*, and *trnK-UUU*, all with 72 exons. Among them, the length of exon I of the three genes was 37 bp, whereas that of exon II was 35 bp (Table 2).

### 3.2. Analysis of the Codon Usage Profiles

**Figure 2:**
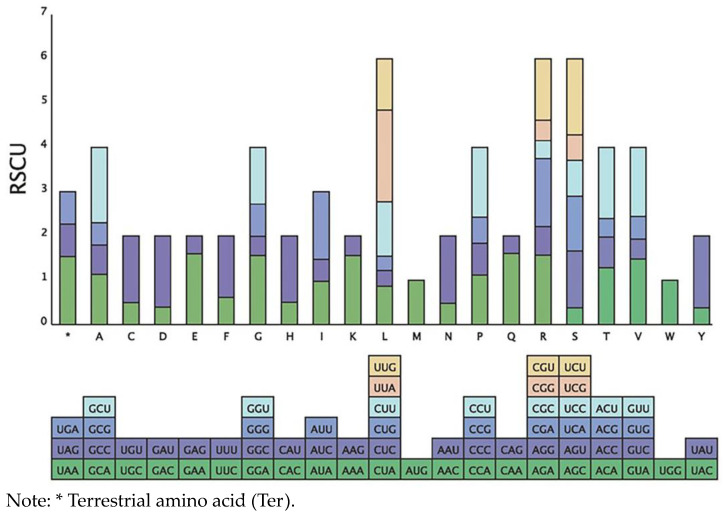
Relative synonymous codon usage of amino acids of *K. ewersmanniana.* The blocks underneath stand for different codon encoding amino acids. The columns on the top depict the sums of RSCU values of the 20 amino acids.

According to codon analysis, we detected 22,696 codons in *K. ewersmanniana*, encoding 20 amino acids (Figure 2, Table 3). Of these, 2395 codons encoded leucine (Leu), accounting for 10.55% of the total codons, exhibiting the highest coding rate. However, only 253 codons encoded cysteines, accounting for the smallest proportion (1.11%). According to the RSCU analysis, 30 codons had RSCU > 1, implying overrepresentation of these codons, whereas 32 codons had RSCU < 1, implying that these codons were used less frequently. In Leu, the UUA had the largest RSCU value, at 2.07, and the GUG encoding Leu had the lowest at 0.32. Moreover, 29 codons had bases ending in A/U, whereas the one remaining codon ended in G. The proportion of codons ending in A/U was 96.67%. Consequently, *K. ewersmanniana* favors codons ending in A/U.

### 3.3. Repeat Analysis

**Figure 3:**
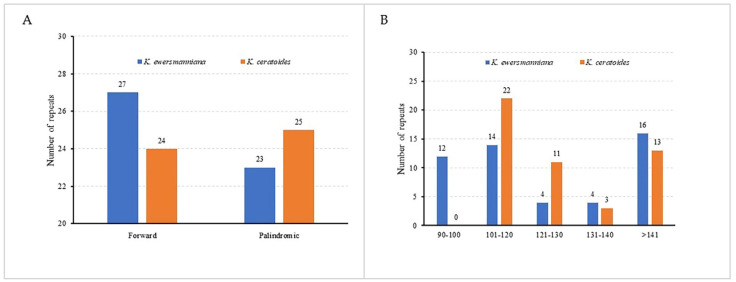
Analysis of tandem repeat sequences in the plastomes of two *Krascheninnikovia* plants. (**A**) type of scattered repeats; (**B**) length of scattered repeats.

**Figure 4:**
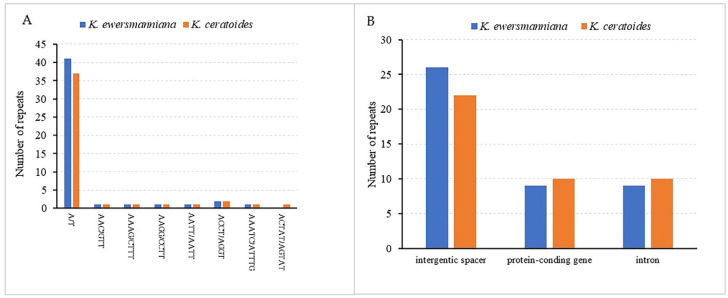
Analysis of SSRs in the chloroplast genome of two *Krascheninnikovia* plants. (**A**) Frequency of identified SSR motifs; (**B**) location distribution of all SSR motifs.

Repeat sequence analysis performed on the genomes of two plants of *Krascheninnikovia* (*K. ewersmanniana* and *K. ceratoides*) detected two different types of scattered repetitive sequences: palindromic (P) and forward (F) (Figure 3A). The number of scattered repetitive sequences, arranged according to length, differed significantly between the two *Krascheninnikovia* species (Figure 3B). A total of 48 SSRs sequences were identified in the chloroplast genome of *K. ewersmanniana*, encompassing seven sequence types. Among them, mononucleotide repeats were the dominant sequence (all A/T), with 41 sequences, accounting for 85.42%. Similar SSRs were found in chloroplast genomes of two *Krascheninnikovia* plants. The total number of SSRs in the *K. ceratoides* chloroplast genome was 45, encompassing eight sequence types. Among them, 37 were single nucleotide repeats (A/T). ACTAT/AGTAT was the only different repeat sequence type identified in *K. ceratoides* (Figure 4A; Table S3). Most of the SSRs in both species were distributed in the intergenic spacer region (26 in *K. ewersmanniana* and 22 in *K. ceratoides*), accounting for more than 50% of all SSRs (Figure 4B). The number of SSRs distributed in the intergenic spacer, PCG, and intron regions was 22, 10, and 10, respectively (Figure 4B). There are 48 SSRs located in the intergenic spacer, and 19 SSRs had introns (Table S4).

### 3.4. Comparison of the Chloroplast Genomes of Seven Amaranthaceae Plants

**Figure 5:**
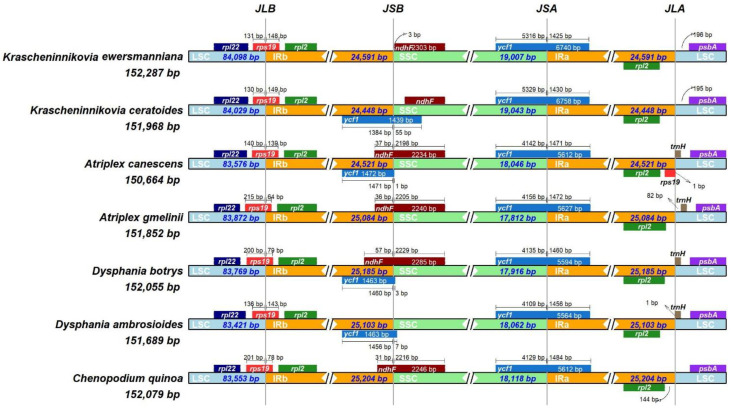
Comparative analysis of four boundary regions in the chloroplast genomes of seven Amaranthaceae plants.

We compared the IR boundary regions and locations of the adjacent genes of *K. ewersmanniana* and six other species of Amaranthaceae (Figure 5). The chloroplast genome structure of *K. ewersmanniana* was highly conserved; however, structural changes were observed in the IR boundary regions. The *rps19* gene exhibited variable expansion at the LSC/IRb regional junction in seven species, including *K. ewersmanniana*, *K. ceratoides*, *Atriplex canescens*, and *Dysphania ambrosioidis*. Among these species, expansion ranged from 139–149 bp in four species and 64–79 bp in three species (*Atriplex gmelinii*, *C. quinoa*, and *Dysphania botris*). The *ndhF* gene exhibited variable contraction or expansion at the IRb/SSC region junction in five species: *K. ewersmanniana*, *A. canescens*, *A. gmelinii*, *D. botrys*, and *C. quinoa*. Except for *K. ewersmanniana*, which exhibited contraction of 3 bp, expansion of 31–57 bp was observed in the other species. Except for *K. ewersmanniana* and *C. quinoa*, the *ycf1* gene was expanded at the IRb/SSC region junction, with a range of 1–55 bp. In all seven species, *ycf1* showed similar expansion at the SSC/IRa region junction. The *psbA-trnH* gene exhibited variable expansion at the IRA/LSC region junction in all species except *K. ewersmanniana*, *K. ceratoides*, and *C. quinoa*. Contraction of 82 bp and 1 bp was observed in *A. gmelinii* and *D. ambrosioides*, respectively, and *rpl2*-*rps19* contraction of 1 bp was only observed at the IRa/LSC region junction in *A. canescens*.

**Figure 6:**
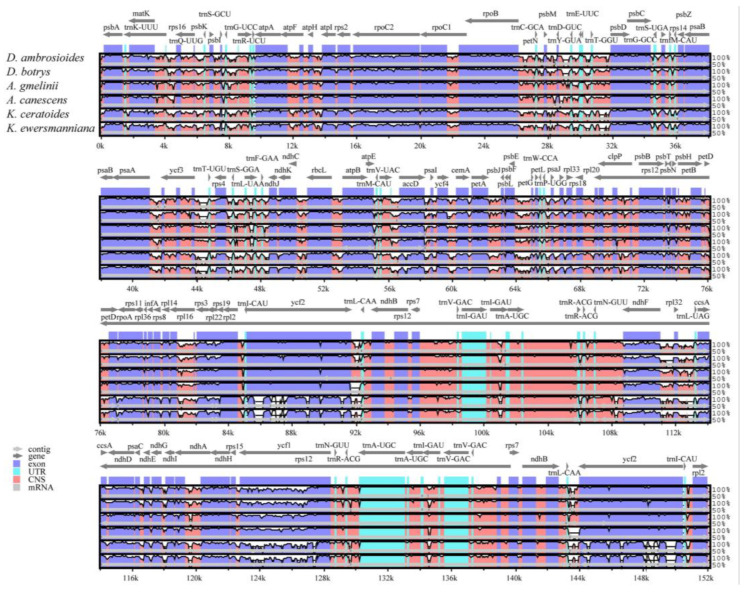
Global alignment of seven chloroplast genomes using *C. quinoa* as a reference. Horizontal axis represents coordinates in the chloroplast genome; vertical axis indicates the average percentage sequence similarity in the aligned regions from 50% to 100%.

The comparison showed similar chloroplast genome sequences for the seven species; however, the variation of non-coding region was significantly higher than that of coding region. PCGs such as *ycf3* and *rpl16* showed significant variation. The regions between genes with a high degree of variation included *psbA*-*trnK-UUU*, *rps2-rpoC2*, *atpI*-*atpF*, *rpl32*-*ccsn*, and *dhB*-*tmL*-*CAA* (Figure 6).

**Figure 7:**
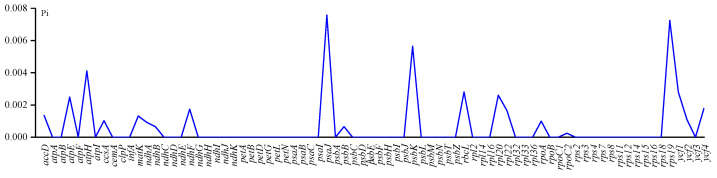
Nucleotide polymorphisms in chloroplast genome of two *Krascheninnikovia* plants. Horizontal and vertical axes show the name of the gene and Pi value, respectively.

Analysis of the nucleotide diversity of the two *Krascheninnikovia* species revealed average Pi values of 0–0.00758 (Figure 7). Three highly variable regions were identified with Pi > 0.005, including *psaJ*, *psbK*, and *psbK*; these sites may contain more rapidly evolving site information and show potential as molecular markers.

### 3.5. Phylogenetic Analysis

**Figure 8:**
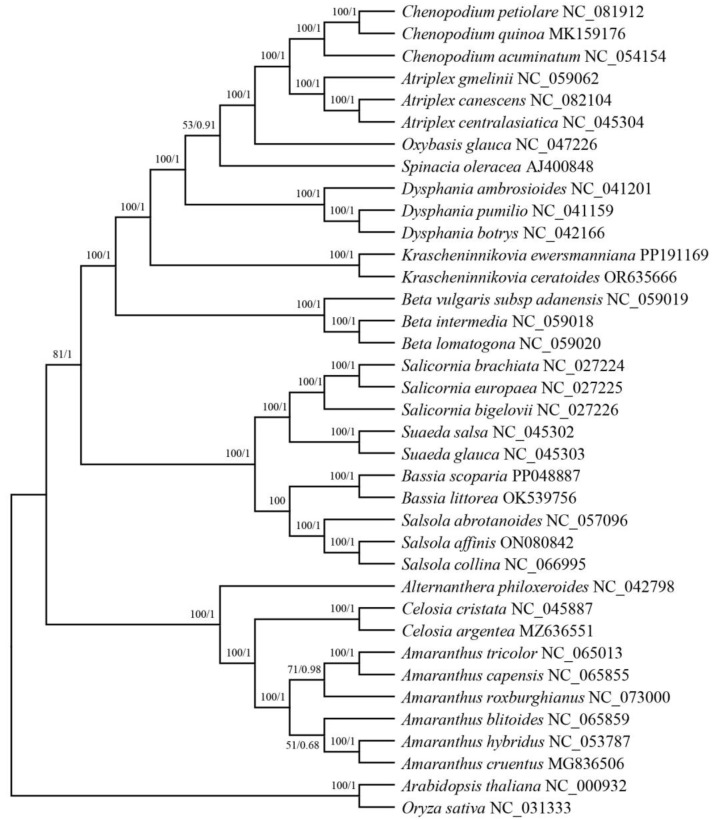
Phylogenetic tree of *K. ewersmanniana* with 34 other representative Amaranthaceae species. *A. thaliana* and *O. sativa* were selected as outgroups. The maximum likelihood and Bayesian tree were determined based on shared protein-coding genes. Maximum likelihood bootstrap support values/Bayesian posterior probabilities are shown for each node.

To determine the phylogenetic position of *K. ewersmanniana*, we extracted and analyzed the shared PCGs of 35 representative Amaranthaceae species (Figure 8) with *O. sativa* and *A. thaliana* as outgroups. A total of 16 species of *Chenopodium*, *Atriplex*, and *Beta* were grouped into a single cluster. The 10 species of *Salicornis*, *Suaeda*, *Bassia*, and *Salsola* were incorporated into one group. Nine species of *Ostosia*, *Celosia*, and *Amaranth* were incorporated into another group. Each genus of plants, all of which are monophyletic, was clustered together. *Alternanthera philoxeroides* was located in the bottom of the phylogenetic tree and is the earliest isolated species among the 35 species in the Amaranthaceae family. *Krascheninnikovia* was located in the middle of the phylogenetic tree. *K. ewersmanniana* and *K. ceratoides* form a separate clade, which was most closely related to two other clades, comprising, respectively, *D. ambrosioides*, *D. botrys*, *D. pumilio*, and *Beta vulgaris*, *Beta intermedia*, *Beta lomatogona*, *Atriplex* (*A. gmelinii* and *A. canescens*), and *Chenopodium* (*C. petiolare* and *C. quinoa*). These clades were located at the top of the phylogenetic tree and are the most recently isolated of the 35 species of the Amaranthaceae family.

## 4. Discussion

The Amaranthaceae family, formerly also named Chenopodiaceae, harbors 80 genera and approximately 2500 species [27], including *Krascheninnikovia*, earlier referred to as *Ceratoides* or *Eurotia*, whose taxonomic position remains unclear. Prior studies have focused on the botanical, biological, and ecological characteristics of this plant [43,44,45]. With the development of sequencing technology, more complete cp genomes have been reported. According to incomplete statistics, approximately 86 complete genomes of the Amaranthaceae family were deposited in the National Center for Biotechnology Information (NCBI, https://www.ncbi.nlm.nih.gov/ (accessed on 22 April 2024) [27,46,47,48,49,50]. However, merely one *Krascheninnikovia* species, *K. ceratoides,* was reported. Genetic and phylogenetic characteristics, therefore, have been significantly lacking for the *Krascheninnikovia* species. *K. ewersmanniana* is a major species of high-quality wild forage grass, providing wind-resistance and sand-fixation for the vegetation in northern Xinjiang. It plays a vital role in vegetation restoration and ecological construction of the desert steppe in Xinjiang. To construct the phylogeny and lay a solid foundation for the evolution of *Krascheninnikovia* and the Amaranthaceae family, the *K. ewersmanniana* complete cp genome was assembled and annotated. The results depict that the chloroplast genome length of *K. ewersmanniana* is 152,287 bp and 128 genes, similar to that of *Chenopodium acuminatum* Willd. (152,200 bp, 113 genes) [51], *Chenopodium album* (152,200 bp, 110 genes) [52], and Amaranthaceae (149,726–153,474 bp, according to NCBI). Their complete cp genomes were all typical tetrad structures and composed of SSC, LSC, and two IRs. Overall, the basic features of the *K. ewersmanniana* chloroplast genome were consistent with those of most Amaranthaceous species, especially for congeners such as *K. ceratoides*, indicating that chloroplast genome size and structures of *Krascheninnikovia* species are highly conserved. The GC content in the IR region was higher than that in the LSC and SSC regions, which may be attributed to the presence of GC-rich genes in the IR region.

Repetitive sequences were the main force of chloroplast genome repeat, deletion, and rearrangement which resulted in the evolution of chloroplast [53]. The two tandem types identified in the *K. ewersmanniana* chloroplast genome were forward (F) and palindromic (P), which is consistent with those of *K. ceratoides*, *C. album*, and *B. vulgaris* [46,54]. Only one tandem type was detected in the chloroplast genomes of *spinach* and *quinoa*, both of which are F-types [55]. These results also differ from those of *Carthamus* (Asteraceae) species [27]. Thus, a correlation exists between the genetic relationships among species and the type and number of repeats. DNA molecular markers are useful tools widely applied in genetic structure analysis, identification of generational relations, and evolution of species. Being highly polymorphic and codominant, SSR markers were given more prominence than RAPD, ISSR, and other markers [56]. In another study, 48 SSRs were detected in *K. ewersmanniana* cp genomes, and mononucleotide A/T as a repeat unit was dominant. Compared with *K. ceratoides*, there was no pentanucleotide [25]. Chloroplast SSR markers can greatly facilitate species detection since they are maternally inherited. For example, a difference only in SSRs (ACTAT/AGTAT) found in the cp genome of two plants allowed for the differentiation and identification of species. Whether this hypothesis is true requires further validation via publication of more cp genomes along with development of SSR molecular markers.

DNA barcoding has been an effective tool to carry out species identification [57], and greatly promoted the development of species classification and phylogeny [58]. DNA barcoding research in higher plants mainly focuses on the chloroplast and nuclear genomes. According to the Consortium for the Barcode of Life, Chloroplast DNA fragments such as *psbA-trnH* and ribosomal DNA fragments such as ITS are widely used DNA barcodes [59]. In the literature, several variations in hotspots, such as *psbA*-*trnK-UUU*, *rps2-rpoC2*, *atpI*-*atpF*, *rpl32*-*ccsn*, and *dhB*-*tmL*-*CAA* and genes, *psaJ*, *psbK*, and *psbK* have been elucidated. Nevertheless, *accD*-*psaI*, *ndhF*-*trnL*, *petA*-*psbJ*, *psbF*-*petL*, *trnC*-*psbM*, *trnS*-*trnG*, and *ycf2*-*trnL* and PCGs such as *accD*, *matK*, *ndhF*, *ndhK*, *ycf1*, and *ycf2* are those with significant variation [25]. Hence, further developing and screening structural variations in the chloroplast genome of *K. ewersmanniana* could be applied to species identification and phylogenetic development for *K. ewersmanniana* with its related species.

The main reason for the structural variation of the chloroplast genome is the variation of IR/LSC boundaries, and its contraction and expansion play key roles in the evolution of plant chloroplast DNA. Compared with that in other species, genetic structural changes in *K. ewersmanniana* occurred in all IR boundary regions. Genes and regions with high degrees of variation were identified and validated as potential molecular markers.

The phylogenetic tree based on chloroplast genome construction showed that 35 species of Amaranthaceae plants were clustered into one group. *K. ewersmanniana* and *K. ceratoides* were grouped together with 100% bootstrap values, and were most closely related to the clade of *Atriplex*, *Chenopodium*, and *Dysphania*, species with 100% bootstrap values; this is consistent with previous research results on the comparative analysis of *K. ceratoides* chloroplast genomes [25]. Atriplex and Dysphania were inserted in the Chenopodium, along with Krascheninnikovia, together divided into Tribe Chenopodieae [60]. Among Amaranthaceae, Liu et al. [25] found that *Suaeda* had the closest phylogeny to *Atriplex. Atriplex* and *Suaeda* formed different topological structures with other genera in this study. Determining the taxonomy based on morphology of *Krascheninnikovia* species has been difficult due to their characteristic transition from entomophily to anemophily and minor flowers. A comprehensive analysis of the morphology has not yet been attained due to lack of research at the molecular level, and the identification and taxonomy of *Krascheninnikovia* and even Amarathaceae are therefore yet to be elucidated.

## 5. Conclusions

To sum up, our study for the first time assembled the chloroplast genome of *K. ewersmanniana*, a plant that is widely distributed in the Xinjiang desert and has important ecological and economic value. This research not only enriches existing molecular biology data for this species, but also expands available genomic resources. In addition, we reported the evolutionary relationship between *K. ewersmanniana* and other species of Amaranthaceae. The results of the phylogenetic tree analysis suggested that *K. ewersmanniana* and *K. ceratoides*, along with *D. ambrosioids*, *D. pumilio*, and *D. botrys*, form a closely related monophyletic group. The phylogenetic insights gained from this study can help address future taxonomic and nomenclature challenges associated with *K. ewersmanniana*. In addition to expanding the available genetic resources of the *Krascheninnikovia* species, this work provides a scientific basis for future research on population genetic diversity and the sustainable utilization of *K. ewersmanniana* resources.

## Figures and Tables

**Table 1:** Gene functional classification and composition of the *K. ewersmanniana* chloroplast genome.

Gene Category	Gene Group	Gene Name
Photosynthesis	Photosystem II	*psbZ*, *psbA*, *psbT*, *psbB*, *psbN*, *psbC*, *psbM*, *psbD*, *psbL*, *psbE*, *psbJ*, *psbK*, *psbF*, *psbI*, *psbH*
	Photosystem I	*psaJ*, *psaB*, *psaI*, *psaC*, *psaA*
	Rubisco	*rbcL*
	Cytochrome b/f complex	petD *, petB *, *petL*, *petA*, *petN*, *petG*
	ATP synthase	atpF *, *atpI*, *atpA*, *atpE*, *atpB*, *atpH*
	NADH-dehydrogenase	ndhB *(2), ndhA *, *ndhI*, *ndhD*, *ndhC*, *ndhE*, *ndhG*, *ndhF ndhH*, *ndhK*, *ndhJ*
Self-replication	rRNA genes	*rrn4.5S(2)*, *rrn23S(2)*, *rrn5S(2)*, *rrn16S(2)*
	Large subunit of ribosome	rpl16 *, *rpl14*, *rpl2(2)*, *rpl22*, *rpl36*, *rpl20*, *rpl33*, *rpl32*
	DNA-dependent RNA polymerase	rpoC1 *, *rpoB*, *rpoC2*, *rpoA*
	tRNA genes	trnA-UGC *(2), trnI-GAU *(2), trnV-UAC *, trnL-UAA *, trnR-UCU *, trnK-UUU *, *trnI-CAU(2)*, *trnfM-CAU*, *trnL-CAA(2)*, *trnW-CCA*, *trnN-GUU(2)*, *trnT-GGU*, *trnS-GGA*, *trnR-ACG(2)*, *trnS-UGA*, *trnV-GAC(2)*, *trnC-GCA*, *trnS-GCU*, *trnD-GUC*, *trnQ-UUG*, *trnY-GUA*, *trnT-UGU*, *trnM-CAU*, *trnP-UGG*, *trnL-UAG*, *trnF-GAA*, *trnG-UCC*, *trnG-GCC*, *trnE-UUC*
	Small subunit of ribosome	rps12 *(2), rps16 *, *rps8*, *rps4*, *rps19*, *rps14*, *rps7(2)*, *rps15*, *rps11*, *rps2*, *rps18*, *rps3*, *rps12*
Other genes	Translation initiation factor	*infA*
	C-type cytochrome synthesis gene	*ccsA*
	Protease	clpP **
	Acetyl-CoA carboxylase	*accD*
	Maturase	*matK*
	Envelope membrane protein	*cemA*
Unknown	Conserved open reading frames	ycf3 **, *ycf2(2)*, *ycf4*, *ycf1*

Note: * only one intron gene; ** two intron genes; gene (2): indicates the presence of two copies of the gene.

**Table 2:** Length of exons and introns of split genes in the chloroplast genome of *K. ewersmanniana*.

Gene	Strand	Start	End	Exon I (bp)	Intron I (bp)	Exon II (bp)	Intron II (bp)	Exon III (bp)
*ycf3*	−	41,858	43,885	124	779	230	805	90
*clpP*	−	69,732	71,733	71	811	292	603	225
*trnK-UUU*	−	1470	4034	37	2493	35		
*rpl16*	−	80,996	82,458	9	1055	399		
*ndhA*	+	118,491	120,536	552	954	540		
*petB*	+	74,655	76,137	6	835	642		
*rps12*	−	139,808	140,608	232	543	26		
*ndhB*	+	92,771	94,970	777	667	756		
*rps12*	+	95,778	96,578	232	543	26		
*trnA-UGC*	−	134,254	135,153	37	827	36+		
*ndhB*	−	141,416	143,615	777	667	756		
*petD*	+	76,359	77,560	8	719	475		
*trnI-GAU*	−	135,224	136,227	37	932	35		
*rps16*	−	4713	5913	100	907	194		
*trnV-UAC*	−	51,060	51,751	38	616	38		
*trnI-GAU*	+	100,159	101,162	37	932	35		
*atpF*	−	11,200	12,593	145	839	410		
*trnL-UAA*	+	46,709	47,436	39	639	50		
*trnA-UGC*	+	101,233	102,132	38	827	35		

Note: + positive strand; − negative strand.

**Table 3:** Codon numbers for *K. ewersmanniana* chloroplast PCGs.

Codon	Count	Codon	Count	Codon	Count	Codon	Count
UAA	40	GGC	168	AUG	501	AGU	353
UAG	19	GGG	280	AAC	253	UCA	340
UGA	19	GGU	492	AAU	796	UCC	224
GCA	363	CAC	132	CCA	265	UCG	157
GCC	211	CAU	392	CCC	169	UCU	472
GCG	160	AUA	626	CCG	140	ACA	381
GCU	544	AUC	320	CCU	374	ACC	205
UGC	63	AUU	982	CAA	655	ACG	124
UGU	190	AAA	978	CAG	162	ACU	476
GAC	181	AAG	280	AGA	338	GUA	462
GAU	736	CUA	344	AGG	141	GUC	140
GAA	1033	CUC	144	CGA	331	GUG	160
GAG	260	CUG	128	CGC	88	GUU	486
UUC	409	CUU	490	CGG	99	UGG	381
UUU	933	UUA	827	CGU	300	UAC	155
GGA	598	UUG	462	AGC	104	UAU	660

## Data Availability

The datasets presented in this paper can be found in online repositories. The names of the repositories and their accession number(s) are available at https://www.ncbi.nlm.nih.gov/ (accessed on 22 April 2024).

## Supplementary Materials

The following supporting information can be downloaded at: https://www.mdpi.com/article/10.3390/genes15050546/s1, Table S1: Accession number and sampled chloroplast genomes obtained from GenBank; Table S2: Characteristics of the chloroplast genome of *K. ewersmanniana*; Table S3: Identified SSRs motifs in the chloroplast genomes of two *Krascheninnikovia* species; Table S4: SSR locations in the chloroplast genomes of two *Krascheninnikovia* species.
